# CD19 CAR-T expressing PD-1/CD28 chimeric switch receptor as a salvage therapy for DLBCL patients treated with different CD19-directed CAR T-cell therapies

**DOI:** 10.1186/s13045-021-01044-y

**Published:** 2021-02-16

**Authors:** Yun Liang, Hui Liu, Zheming Lu, Wen Lei, Chaoting Zhang, Ping Li, Aibin Liang, Ken H. Young, Wenbin Qian

**Affiliations:** 1 Department of Hematology, The Second Affiliated Hospital, College of Medicine, Zhejiang University, Hangzhou, Zhejiang People’s Republic of China; 2 Key Laboratory of Carcinogenesis and Translational Research (Ministry of Education/Beijing), Laboratory of Biochemistry and Molecular Biology, Peking University Cancer Hospital and Institute, Beijing, 100142 People’s Republic of China; 3 Department of Hematology, Tongji Hospital of Tongji University, Shanghai, People’s Republic of China; 4 Hematopathology Division and Department of Pathology, Duke University Medical Center and Cancer Institute, Durham, NC USA; 5 Institute of Hematology, Zhejiang University, Hangzhou, 31003 Zhejiang People’s Republic of China; 6 National Clinical Research Center for Hematologic Diseases, the First Affiliated Hospital of Soochow University, Suzhou, Jiangsu, People’s Republic of China

**Keywords:** DLBCL, CAR T cell therapy, PD-1/CD28 chimeric switch receptor, Salvage therapy

## Abstract

CD19-targeted chimeric antigen receptor T (CAR T) cell therapy is a promising option to treat relapsed/refractory diffuse large B-cell lymphoma (R/R DLBCL). However, the majority of CAR T-treated patients will eventually progress and require salvage treatment, for which there is no current standard. In this study, we analyzed data from 6 patients with R/R DLBCL who experienced progression following CD19-CAR T therapy, and then received CD19-specific CAR T cells that express a PD-1/CD28 chimeric switch-receptor (CD19-PD-1/CD28-CAR T) as salvage therapy at our institution. After the second infusion of CAR T cells, 3 of 6 patients achieved complete remissions and the duration of the response of responsive patients ranged from 8 to 25 months. One patient showed a stable disease. In contrast, 2/6 patients died on 60 days because of progression disease. Importantly, no severe neurologic toxicity or cytokine release syndrome was observed. These data suggest that CD19-PD-1/CD28-CAR-T cells, a novel anti-CD19 CAR-T cell therapy, elicit a potent and durable anticancer response, and can be used in the post-CD19-CAR T failure setting.

**Supplementary Information:**

The online version contains supplementary material available at 10.1186/s13045-021-01044-y.

**To the Editor:**

CD19-specific CAR T cell therapy has significantly improved the outcome of patients with R/R DLBCL, resulting in durable remissions in approximately 40% of heavily pretreated patients. Despite these encouraging results, nearly half of the patients could not achieve durable response after CD19-CAR T therapy and a significant proportion of patients will eventually relapse and develop treatment-refractory, fatal disease [1–4]. Recently, Spiegel et al. [5] reported on outcomes of large B-cell lymphoma patients who experienced progression following CD19-CAR T (Axicabtagene ciloleucel, axi-cel). The results showed that median overall survival (OS) from date of progression disease was 180 days (95% CI 105–242). Until now, there is no recommended therapeutic schedule for this fatal disease.

Six patients who relapsed or were refractory to CD19-CAR T therapy have been treated with CD19-PD-1/CD28-CAR-T, a novel anti-CD19 CAR-T cell therapy [6], on compassionate-use basis at our institution between January 2018 and August 2019. This retrospective study was approved by the Ethics Committee of the Second Affiliated Hospital, Zhejiang University (Hangzhou, China) and conducted in accordance with the principles of the Declaration of Helsinki. As of October 1, 2020, the median follow-up time was 14 months. Six consecutive patients were enrolled. Patients ranged from 47 to 63 years of age and had received prior CD19-CAR T therapy with CD28-based or 4-1BB-based CAR T cells. Three of the six patients were refractory to the first CAR T treatment, and two patients had partial remission (PR) with response duration of 3 and 4 months, respectively. One patient had a CR duration of 30 months before relapse (Table 1 and Fig. 1a). CD19-PD-1/CD28-CAR-T cells manufactured from the leukapheresed or cryopreservated autologous peripheral blood mononuclear cells were successful for all 6 patients. After failure of first CAR T therapy, patients received conditioning chemotherapy containing cyclophosphamide 500 mg/m^2^ and fludarabine 30 mg/m^2^ daily on days -5 to -3, and followed by a single intravenous infusion of CD19-PD-1/CD28-CAR-T as a salvage treatment for refractory or relapsed disease. The therapeutic doses of CAR T range from 0.32 × 10^6^ to 4 × 10^6^/kg of body weight. A total of 90 related adverse events occurring within 30 days of CAR-T infusion were recorded between grade 1 and 4 (Additional file 1: Table S1). Overall, 3/6 patients experienced grade 1 CRS, Patient 2 experienced grade 2 CRS, and Patient 4 and Patient 6 had both grade 2 CRS and grade 3 ICANS (immune effector cell‐associated neurotoxicity syndrome). Serum cytokine levels were detected in all patients during the first month following second CAR-T therapy (Additional file 2: Figure S1). IL-6, IL-4, IL-2, and TNFα were elevated in Patient 1. Patient 4 who experienced both grade 2 CRS and grade 3 ICANS exhibited increased levels of IL-6, IL-4, IL-2, IL-17A, and IFNγ, but such increased levels were not observed in Patient 6. Four cases of CRS resolved fully by supportive treatment while the 2 patients suffered from both CRS and ICANS resolved completely after treatment with supportive care, tocilizumab and glucocorticoids. The response was evaluated with FDG-PET-CT at 3 months after infusion (Fig. 1b), according to the International Working Group Response Criteria for Malignant Lymphoma. As shown in Table 1 and Fig. 1, three of six patients achieved a CR, and one patient showed stable disease. In contrast, 2/6 patients died on 60 days because of progression disease. Two of three patients achieving CR maintained ongoing response on the date for the last visit. But, another one relapsed within 8 months and eventually died 12 months after CAR T treatment. The presence of CAR-T cells in patients' blood was monitored by qPCR. The number of blood CAR^+^ cells peaked within 2 weeks after infusion. However, peak blood CAR^+^ cell numbers did not differ significantly between patients with response and those without response. Interestingly, CAR^+^ cell numbers dropped rapidly after peaking but increased significantly by day 540 after CAR T treatment in Patient 3 who achieved durable remission with long, treatment-free interval (Fig. 1c).

**Table 1:** Clinical characteristics and post-CAR T outcomes

Patient Number	1	2	3	4	5	6
Age (years)	51	51	53	47	56	63
Sex	Male	Male	Female	Female	Female	Male
Diagnosis/subtype	TFL	DLBCL/GCB	DLBCL/non-GCB	HGBL (Triple hit)	DLBCL/non-GCB	DLBCL/non-GCB
ECOG PS^1^	1	3	1	3	3	1
Prior therapy	R-CHOP; 2-HyperCVAD-A	R-CHOP; R-BEAM + ASCT	R-CHOP; R-GDP; R2-COP + mitoxantrone; BEAM + ASCT	CHOP; R- EPOCH; R-DA-EPOCH; DHAP	R- EPOCH; R-GeMox; ICE^2^ R-COP^2^; Ibrutinib^2^	R-CHOP; R-GDP; R2-MINE; R-DHAP
Disease Stage^1^	4	3	4	4	4	3
B Symptom^1^	A	A	B	A	A	A
PD-L1^1^	20%	25%	Negative	Negative	Negative	Negative
Ki-67^1^	80%	50%	80%	100%	40%	80%
IPI Score^1^	2	3	3	3	4	2
*First CAR-T*						
Costimulatory domain	4-1BB	4-1BB	CD28	CD28	4-1BB	4-1BB
Dose (10^6^/kg)	0.50	1.00	0.88	1.78	4.00	2.00
Response	PR	PD	PD	PD	PR	CR
DOR (Month)	4	NA	NA	NA	3	30
*Second CAR-T*						
ECOG PS	2	4	1	4	3	1
Dose (10^6^/kg)	0.32	0.63	0.50	2.90	4.00	2.00
Response	PD	ED	CR	ED	CR	CR
DOR (Month)	NA	NA	25	NA	8	14
PBMC Source	Leukapheresis	Cryopreservation	Cryopreservation	Cryopreservation	Leukapheresis	Leukapheresis
Time for CAR-T culture (Day)	15	14	14	13	14	12
Naïve T (%)	1.89	29.85	25.35	51.9	31.25	28.64
TCM T (%)	60.48	26.84	40.49	42.89	47.97	13.66
TEM T (%)	1.14	21.35	23.26	3.81	13.61	13.16
TEMRA T (%)	36.49	21.96	10.90	1.40	7.17	44.55

ASCT, autologous stem cell transplant; CHOP, cyclophosphamide, adriamycin, vincristine, prednisone; COP, cyclophosphamide, vincristine, prednisone; EPOCH, etoposide, prednisone, vincristine, cyclophosphamide, doxorubicin; BEAM, carmustine, etoposide, cytarabine, melphalan; GDP, gemcitabine, dexamethasone, cisplatin; GEMOX, gemcitabine, oxaliplatin; ICE, ifosfamide, carboplatin, etoposide; HyperCVAD, cyclophosphamide, vincristine, doxorubicin, dexamethasone; DHAP, dexamethasone, high dose cytarabine, cisplatin; ICE, ifosfamide, carboplatin, etoposide; MINE, mesna, ifosfamide, mitoxantrone, etoposide; R, rituximab; R2, rituximab combined with lenalidomide; DLBCL, diffuse large B cell lymphoma; ECOG PS, Eastern Cooperative Oncology Group performance status; IPI, International Prognostic Index; GCB, germinal center B cell; TFL, transformed follicular lymphoma; DLBCL, diffuse large B-cell lymphoma; HGBL, high grade B-cell lymphoma; Triple hit, MYC, BCL2 and BCL6 rearrangements. DOR, Duration of Response; TCM, T cells with central memory; TEM, T cells with effector memory; TEMRA, terminally differentiated effector T cells

^1^These data were collected before first CAR-T

^2^The chemotherapy regimens were conducted after first CAR-T

**Fig. 1:**
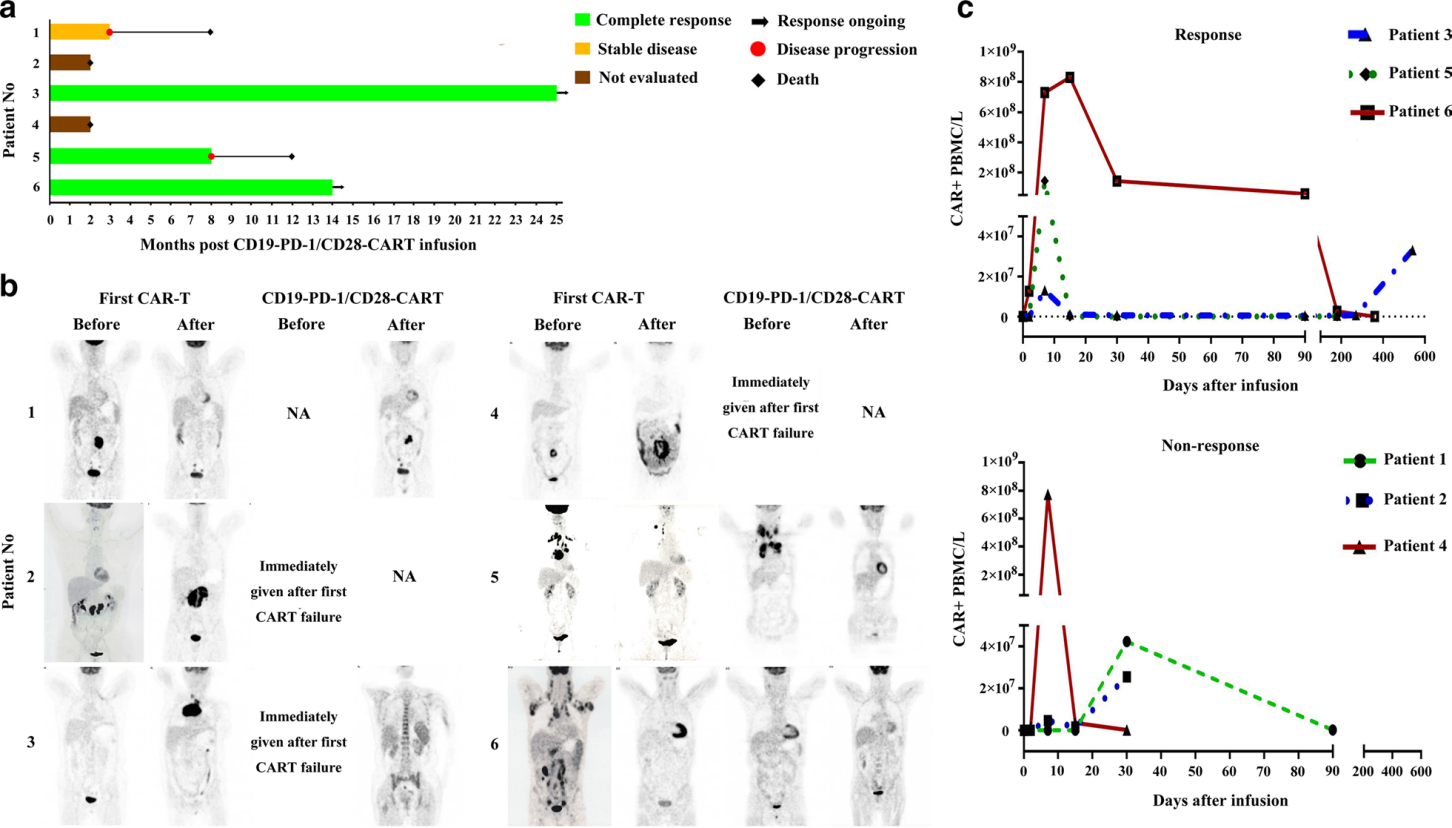
CD19-PD-1/CD28-CAR T induced durable clinical responses in R/R large B-cell lymphoma patients after failure of CD19-CAR T therapy. **a** Treatment response of each patient and the duration of response post-infusion with CD19-PD-1/CD28-CART. **b** Representative PET-CT scans at baseline and 90 days post first CAR-T infusion and salvage CAR T therapy. **c** The absolute number of CAR + peripheral blood mononuclear cells in patients who achieved clinical responses or non-responses were quantified by PCR

Treatment options are limited for DLBCL patients when disease fails to respond to or relapses after CD19 CAR-T cell therapy. Novel therapies with different mechanisms of actions are critical to improve unmet clinical needs and the outcome of these patients. Alternative CD19-specific CAR-T product may be an active salvage therapy although no clinical trials have defined the optimal approach. A recent study evaluated the efficacy of axi-cel, a CD28 costimulatory-based CD19-CAR T, as salvage therapy after failure of tisagenlecleucel or investigational CD19 CAR-T with 4-1BB costimulation in patients with DLBCL. One of three patients achieved CR, but died 180 days after axi-cel treatment because of progressive disease while two of three patients were refractory [7]. In another clinical study, second infusion of CD19-CAR T was used as salvage treatment after failure of first CD19 CAR T-cell therapy. Of 21 Non-Hodgkin lymphoma (NHL) patients, the overall response rate after the second CAR T was 52% (CR, *n* = 4; PR, *n* = 7) [8]. In the present study, 3 of 6 patients achieved CRs, 2 of 3 CRs are ongoing, suggesting that CD19-PD-1/CD28-CAR-T elicit a potent and durable anticancer response, and can be used in the post-CD19 CAR T failure setting. However, we did not find an association between the costimulatory domain of CAR T and disease control (Table 1).

CAR T cell efficacy can be enhanced by using engineering strategies to address the challenge relating to T cell exhaustion induced by PD-1/PD-L1 pathway [9–11]. Until now, few cases report rare lymphoma patients who could obtain better efficacy by a combination of CAR-T cell therapy and PD-1 blockade [10, 12]. Recently, we have reported that CD19-PD-1/CD28-CAR T cells exhibited a superior capability of killing PD-L1^+^ B-cell lymphoma cells in vitro and in vivo relative to the prototype, CD19-CAR T cells. We also demonstrated that this therapy had a favorable safety profile and induced durable clinical responses in the patients with PD-L1^+^ R/R DLBCL [6]. An interesting aspect of the current study was that CD19-PD-1/CD28-CAR T was generally well-tolerated and resulted in a high response rate that was durable in R/R large B-cell lymphoma after failure of CD19-CAR T therapy. In conclusion, our data demonstrate the ability to augment CAR T cells targeting CD19^+^ lymphoma by co-expressing a chimeric PD-1/CD28 switch-receptor, and that this therapy has potential as a salvage treatment when first CAR T proves ineffective or resistant.

## Supplementary Information

**Additional file 1: Table S1**. Treatment-emergent adverse events.

**Additional file 2: Figure S1**. Kinetics of serum cytokines. A-H. Fold change of listed serum cytokines obtained from patients at the indicated times was calculated relative to the baseline.

## Data Availability

All data generated or analyzed during this study are included in this paper and its Supplementary files.

## Abbreviations

DLBCL: Diffuse large B cell lymphomas
NHL: Non-Hodgkin lymphoma
PD-1: Programmed cell death 1
PD-L1: Programmed cell death 1 ligand
CAR T: Chimeric antigen receptor T
R/R DLBCL: Relapsed/refractory diffuse large B-cell lymphoma
CRs: Complete remissions
PR: Partial remission
OS: Overall survival
CRS: Cytokine release syndrome
ICANS: Immune effector cell-associated neurotoxicity syndrome
