# Supplementary material for: CD19 CAR-T expressing PD-1/CD28 chimeric switch receptor as a salvage therapy for DLBCL patients treated with different CD19-directed CAR T-cell therapies
Source: J Hematol Oncol. 2021 Feb 16;14:26. doi: 10.1186/s13045-021-01044-y (PMC7885572; doi:10.1186/s13045-021-01044-y)
Supplement: Supplementary file 1 — Additional file 1: Table S1. Treatment-emergent adverse events. [file 13045_2021_1044_MOESM1_ESM.docx]

**Supplementary Table 1. Treatment-emergent adverse events.**

| Event | Any, n (%) | Grade 1, n (%) | Grade 2, n (%) | Grade 3, (%) | Grade 4, (%) |
| --- | --- | --- | --- | --- | --- |
| Fever | 6 (100) | 2 (33.3) | 2 (33.3) | 2 (33.3) |  |
| Chills | 2 (33.3) | 2 (33.3) |  |  |  |
| Weakness | 2 (33.3) |  | 2 (33.3) |  |  |
| Edema | 1 (16.7) | 1 (16.7) |  |  |  |
| Nausea | 4 (66.7) | 3 (50) | 1 (16.7) |  |  |
| Vomiting | 3 (50) | 3 (50) |  |  |  |
| Dyspnea | 1 (16.7) |  | 1 (16.7) |  |  |
| Hypotension | 2 (33.3) | 1 (16.7) | 1 (16.7) |  |  |
| Tremor | 2 (33.3) | 1 (16.7) | 1 (16.7) |  |  |
| Epilepsy | 1 (16.7) |  |  | 1 (16.7) |  |
| Thrombocytopenia | 3 (50) | 1 (16.7) |  | 2 (33.3) |  |
| Anemia | 3 (50) | 3 (50) |  |  |  |
| Neutropenia | 4 (66.7) |  |  | 1 (16.7) | 3 (50) |
| Hypofibrinogenemia | 3 (50) | 2 (33.3) | 1 (16.7) |  |  |
| CRS | 6 (100) | 3 (50) | 3 (50) |  |  |
| ICANS | 2 (33.3) |  |  | 2 (33.3) |  |
